# Divide or bridge? The heterogeneous effects of digital village participation on rural residents’ happiness

**DOI:** 10.3389/fpsyg.2026.1893504

**Published:** 2026-09-18

**Authors:** Zhou Ren, Cancan Zhang, Jingbo Li, MengYuan Yao, Long Fan

**Affiliations:** 1 School of Civil Engineering and Architecture, Nanyang Normal University, Nanyang, China; 2 School of Foreign Languages, Nanyang Normal University, Nanyang, China; 3 Longyou Longqiu Urban and Rural Planning & Design Institute Co., Ltd., Quzhou, China; 4 Henan Urban and Rural Planning and Design Research Institute Co., Ltd., Zhengzhou, China; 5 College of Forestry, Henan Agricultural University, Zhengzhou, China

**Keywords:** credit constraints, digital divide, digital village participation, happiness, social interaction

## Abstract

**Introduction:**

Enhancing rural residents’ happiness is a key objective of China’s rural revitalization strategy.

**Methods:**

Using data from the China Rural Revitalization Survey (CRRS), this study empirically examines the relationship between digital village participation on rural residents’ happiness.

**Results:**

The results show that digital village participation is positively associated with rural residents’ happiness. Dimensional-specific analyses further reveals that digital infrastructure, digital governance, and the digitalization of daily life exert significant positive effects on rural residents’ happiness, whereas the effect of rural economic digitalization is statistically insignificant. The mechanism analysis indicates that digital village participation improves rural residents’ happiness by alleviating credit constraints and increasing social interaction. The heterogeneity analysis reveals that rural residents with higher digital literacy are better able to translate digital participation into gains in happiness, whereas the effect is insignificant among those with lower levels of digital literacy.

**Discussion:**

This study provides empirical evidence for narrowing the rural digital divide and offers policy implications for promoting targeted and differentiated digital village development.

## 1 Introduction

Happiness, as one of the ultimate goals of human development, has long been a central topic in academic discussion. Several studies have examined the determinants of subjective wellbeing. Early research mainly focused on income and economic conditions (La et al., 2021; Stevenson and Wolfers, 2013), whereas more recent studies have expanded their focus to areas such as social capital, institutional quality, and digitalization (Adedeji et al., 2023; Chen and Kühner, 2026; Shiroka-Pula et al., 2023). Despite the abundant research accumulated thus far in the context of the digital transformation wave, the wellbeing of rural populations, especially those in developing countries undergoing rapid transformation, warrant further exploration. In China, rural residents constitute a significant proportion of the population, but they have long lagged behind urban residents in terms of income, public services, and social welfare (Fang et al., 2025a; Ma and Chen, 2020; Yang et al., 2019). Given this situation, China’s recent policy agenda has clearly identified improving people’s wellbeing as an important goal of the modernization process. The rural revitalization strategy is the key institutional framework for achieving this goal. Against this backdrop, understanding the factors influencing the rural residents’ happiness is not only an inherent requirement of rural revitalization but also a crucial indicator for measuring the effectiveness of coordinated urban–rural development.

Since the “Digital Village Strategy” was launched in 2018, digital infrastructure, e-commerce, and online life services have been gradually promoted in rural China, profoundly changing the production and lifestyles of rural residents. However, whether the widespread adoption of digital technology necessarily increases happiness remains an important empirical question. On the one hand, digital technology may reduce information asymmetry, expand market opportunities, and improve access to public services, thereby becoming a “bridge” for rural residents to achieve a better life (Khwidzhilli et al., 2025; Li et al., 2023; Mao et al., 2024). On the other hand, if the benefits of digitalization are distributed unevenly and the participation threshold is too high, the rapid advancement of digitalization may create new “gaps” between urban and rural areas, as well as within rural areas, due to differences in access, digital literacy, and sense of gain (Bai et al., 2023; Hodge et al., 2017; Wang et al., 2025a). This issue concerns not only the effectiveness of digital village policies but also the ethical aspects of rural development in the digital age.

An increasing number of studies have found that the penetration of digital technology into rural areas can enhance the economic wellbeing and rural residents’ happiness by improving livelihood resilience, expanding market access, and upgrading labor skills (Chen et al., 2024; Lyu et al., 2025; Rotz et al., 2019). For instance, Internet use has been found to increase farmers’ income, promote entrepreneurial activities, and alleviate credit constraints (Liu et al., 2025; Wu et al., 2026). Some scholars have also pointed out that the use of information technology can expand rural residents’ social networks, enhance information sharing and mutual assistance, and thereby indirectly improving their subjective wellbeing (Zhou et al., 2023). Furthermore, some studies have directly examined the relationship between digital technology and happiness, finding a U-shaped relationship between rural residents’ digital skills and happiness, with the effect varying significantly across families with different income levels (Zhou et al., 2025). However, some studies indicate that “digital dividends” distribution is uneven. Certain groups, such as the elderly, those with low educational levels, and those lacking digital skills, may not benefit equally from the digitalization process and may even experience a decline in happiness due to an intensified sense of relative deprivation (Li et al., 2026; Wang et al., 2025b; Zou et al., 2025). These contradictory findings suggest that the relationship between digital village participation and happiness may not be simply positive and linear but may instead be influenced by a variety of structural factors, with the “digital divide” at different levels playing a crucial role in this process.

Although an increasing number of studies have examined the relationship between digital technology and rural development, several research gaps remain. First, most studies focus on the impact of individual digital technologies (such as internet use or mobile payment) on specific outcomes (such as income or entrepreneurship), while few take a holistic approach to capturing the multidimensional characteristics of digital village development as a systematic initiative. Second, the mechanism through which digital participation translates into subjective wellbeing, particularly the mediating roles of access to credit and social interaction, lack sufficient empirical verification. Third, although the concept of the digital divide has been extensively studied, how it affects the benefits of digital participation requires further exploration. In particular, how these digital divides affect the effectiveness of digital village participation warrants further exploration.

To fill these gaps, this study utilized nationally representative China Rural Revitalization Survey (CRRS) data to empirically investigate the association between digital village participation and the rural residents’ wellbeing, as well as the potential mediating mechanisms and heterogeneous effects of the three-level digital divide. Through this analysis, this study aims to provide a more systematic and detailed understanding of the complex relationship between rural digital village participation and subjective wellbeing among rural residents in China. Specifically, this study contributes to the existing literature in three ways. First, unlike previous studies that focus on individual digital technologies, this study adopts a multidimensional analytical framework to capture the differentiated associations between the four dimensions of digital village participation and wellbeing. Second, this study empirically identified two mediating pathways: the alleviation of credit constraints and social interaction. These findings provide microlevel evidence for understanding the mechanisms by which digital participation affects wellbeing. Third, it expands the existing literature on the digital divide and systematically examines how the digital divide at the levels of access, skills, and outcomes affects the relationship between digital participation and wellbeing. Thus, this study provides a more detailed understanding of the boundary conditions of the relationship between digital participation and wellbeing in rural China.

The remainder of this study is structured as follows. Section 2 presents a theoretical framework and literature review; Section 3 describes the data sources, variable measurements, and econometric model specifications; Section 4 reports the empirical results; Section 5 provides a discussion; and Section 6 presents the conclusions and policy implications.

## 2 Literature review

### 2.1 Impact of digital village participation on rural residents’ happiness

The widespread adoption of digital technologies has profoundly reshaped the production and lifestyles, thereby exerting complex influences on their subjective wellbeing. Regarding the relationship between digital technology and residents’ happiness, previous studies have examined the issue from multiple perspectives, highlighting the positive effects of digital participation while also considering the potential negative impacts of the digital divide. Most studies have shown that participation in digital village development not only brings economic benefits to rural residents but also enhances their sense of social identity and self-realization (Zou et al., 2025). This psychological empowerment effect is particularly valuable for rural residents who have long been on the periphery of social discourse. This effect helps alleviate the feelings of relative deprivation and improve mental health. Digital transformation in rural areas has significantly enhanced household incomes and development capabilities, expanded social networks, and enabled rural residents to experience greater happiness by improving their abilities and participation in society (Chen et al., 2025). Further research has also revealed that the digital village construction can significantly alleviate the depressive symptoms among the rural elderly (Wan et al., 2025), and improve the accessibility of rural elderly care services (He et al., 2025; Liu et al., 2023). The resulting improvement in service convenience not only saves time and economic costs but also meets rural residents’ expectations for modern lifestyles, thereby increasing their life satisfaction.

However, the positive effect of digital technology on happiness is not unconditional. The digital divide has long been a focus of attention for scholars (Hollman et al., 2021). Significant disparities remain between urban and rural areas in terms of digital infrastructure, digital literacy, and the adaptability of digital content, making it difficult for some rural groups, especially the elderly, those with low education levels, and low-income residents, to participate effectively in the digital society (Yang et al., 2021). For these groups, the introduction of digital technology may not improve wellbeing; instead, it may create new forms of exclusion and frustration due to technological barriers (Rui et al., 2026). Previous studies indicate that the relationship between digital village participation and happiness is neither consistently positive nor universally negative. Instead, the relationship is likely to depend on multiple factors. This study aims to provide additional insights into the relationship between digital village development and wellbeing by adopting a multidimensional framework for digital village participation and systematically examining its relationship with happiness.

### 2.2 Mechanisms through which digital village participation affects rural residents’ happiness

(1) Digital village participation enhances rural residents’ happiness by alleviating credit constraints

Credit constraints are a long-standing structural problem in rural financial markets (Khan et al., 2024). The traditional rural credit market is constrained by factors such as information asymmetry, lack of collateral, and high transaction costs. Consequently, credit supply from formal financial institutions is insufficient, leaving rural residents facing persistent financing difficulties (Khan et al., 2024). Credit constraints not only limit households’ productive investment capacity and income growth potential but also reduces their ability to cope with negative shocks, such as illness and natural disasters, due to the lack of liquidity buffers, thereby exacerbating economic vulnerability and psychological anxiety (Kassouri and Kacou, 2022; Peng et al., 2021). In this context, digital village participation, particularly through the widespread adoption of digital financial services, provides a new technological approach to alleviating credit constraints.

Many empirical studies have found the significant role of digital financial participation in alleviating rural credit constraints. Studies have shown that the widespread adoption of digital payments has significantly expanded the channels through which families obtain informal credit (Wang et al., 2025c). Moreover, farmers participating in e-commerce platforms are more likely to obtain business loans than those who do not (Yu and Xiang, 2021). More importantly, digital finance significantly improves credit accessibility for vulnerable groups that traditional financial institutions struggle to serve, demonstrating its inclusive nature (Yu et al., 2024). Some scholars have also found that improved credit accessibility has enabled rural residents to increase their family income through non-agricultural employment and entrepreneurship (Zhan et al., 2025). Income is a foundation of happiness. An increase in income directly improves residents’ living conditions and consumption capacity and enhances happiness by increasing satisfaction with living standards. Furthermore, inclusive digital finance has effectively stimulated rural residents’ entrepreneurial activities by enhancing service accessibility, improving digital literacy, and increasing household income, thereby promoting inclusive growth and the achievement of sustainable development goals (Agbenyo et al., 2026).

(2) Digital village participation influences rural residents’ happiness by increasing social interaction

Social interaction is a core component of rural residents’ social capital. However, with increasing rural to urban migration, deepening village hollowing, and weakening traditional community ties, social interaction networks in rural areas are at risk of shrinking. The weakening of the social support system directly threatens residents’ happiness (Fang et al., 2025b; Wang and Uekita, 2026). Digital village participation offers new possibilities for reconfiguring and expanding social interactions. Some scholars have found that digital platforms, such as social media, instant messaging tools, and online communities, transcend the limitations of time and space, enabling rural residents to establish and maintain social connections with a wider range of people (Wu and Liu, 2025).

Previous studies have provided empirical evidence of a positive association between digital participation in rural areas and social interaction. The penetration of digitalization has significantly facilitated the expansion of social networks within entrepreneurial households by improving rural residents’ access to information and connectivity. The integration of digital technologies not only enhances households’ perception of market opportunities but also serves as an important intermediary bridge for converting digital resources into entrepreneurial actions (Yang et al., 2023). Furthermore, social media use has significantly reduced communication barriers for the elderly, thereby increasing the frequency and depth of daily interactions with family members and friends and positively affecting informal social participation (He et al., 2020). Moreover, moderate social interaction enhances happiness by providing emotional resonance and a sense of belonging and is a crucial component of the individual wellbeing (Ren et al., 2022). Furthermore, from the perspective of social support theory, social interaction networks are important sources of emotional, informational, and instrumental support (Zhu and Hu, 2023). For rural residents, digitally driven rural transformation has reshaped how this support system operates. For instance, a case study of Kaliabu Village in Indonesia demonstrated that digital participation facilitated new forms of social interaction and mutual assistance among rural practitioners (Fahmi and Sari, 2020). This new interaction model not only enhances the subjective wellbeing but also generates positive external effects for the broader community network.

The theoretical framework are shown in Figure 1.

**Figure 1:**
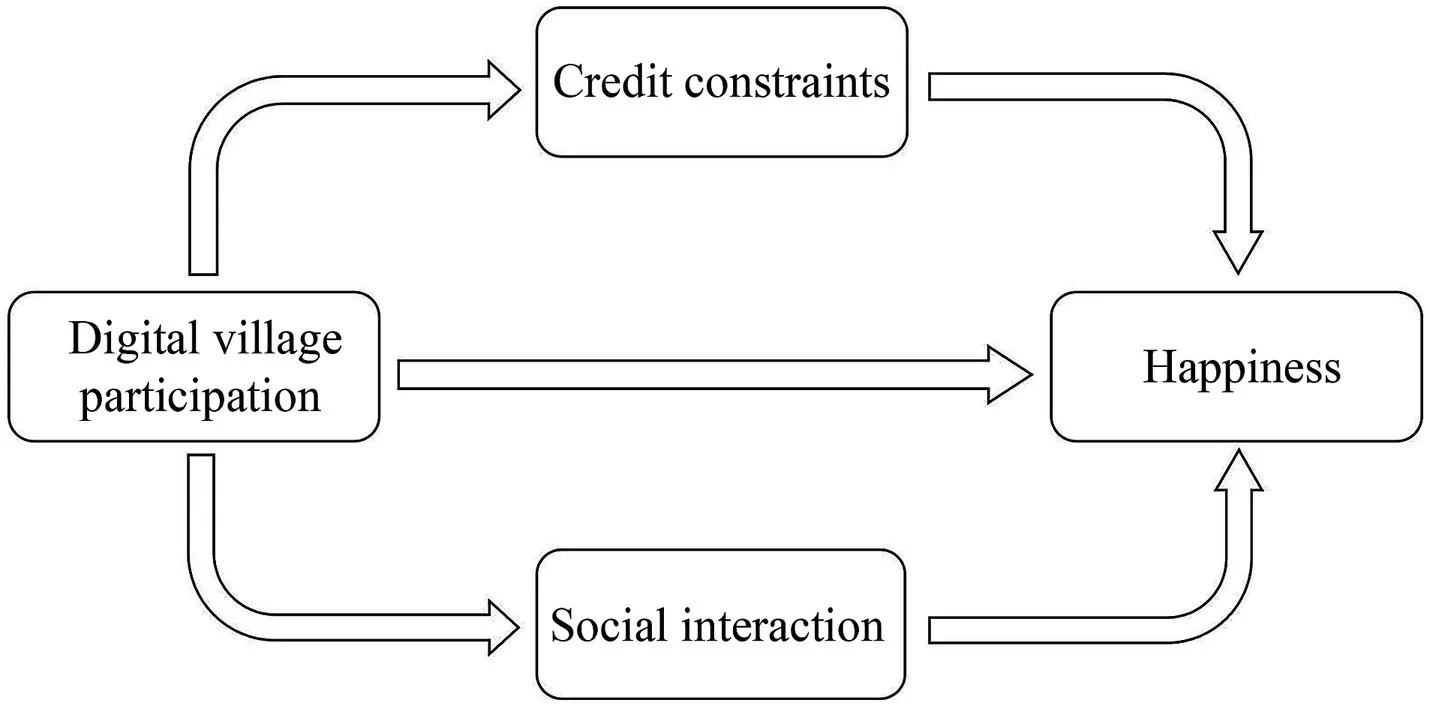
Theoretical framework diagram.

## 3 Methods and data

### 3.1 Data source

The data used in this study were obtained from the China Rural Revitalization Survey (CRRS) conducted by the Rural Development Institute of the Chinese Academy of Social Sciences. The survey adopted a stratified random sampling strategy based on regional economic development levels and geographic characteristics, covering 10 provinces, 50 counties and cities, 156 townships, and 300 villages, with more than 3,800 household questionnaires collected nationwide. The survey collected comprehensive information on the rural population and labor, rural development, household income and expenditure, and social wellbeing, providing a solid empirical foundation for this study. After excluding observations with missing or abnormal values, the final sample comprised 3,126 valid observations.

### 3.2 Variables

#### 3.2.1 Dependent variables

The dependent variable in this study was rural residents’ happiness. Following the existing literature, rural residents’ happiness was measured using respondents’ self-reported happiness scores, an approach that has been widely used and validated in prior research (Ding and Zhang, 2025; Zhao et al., 2024). Specifically, following Zhao et al. (2024), this study used the survey question “Are you happy with your current life?” to measure rural residents’ happiness. Responses were recorded on a five-point Likert scale ranging from 1 to 5, where 1 indicated “very unhappy” and 5 indicated “very happy.” Higher values corresponded to higher levels of happiness.

#### 3.2.2 Core explanatory variable

The core explanatory variable in this study was digital village participation. The existing literature typically evaluates the progress of digital village development along four primary dimensions: digital infrastructure, digital economy, digital life, and digital governance. Following this approach, this study measured household participation in digital village development using four survey items: (1) “How would you assess your household’s internet conditions?”; (2) “Does your household engage in online product transactions?”; (3) “Have you communicated with other villagers on important public affairs through WeChat groups?”; and (4) “Do you use mobile phones or the internet to meet your daily needs?”. A binary indicator was constructed based on these four items. If a respondent reported participation in any of the above activities, the variable was assigned a value of 1, indicating participation in digital village development; otherwise, it was assigned a value of 0. We use binary variables because this study focuses on the extensive margin of digital village participation—that is, whether the household has crossed the threshold from “non-participation” to “any form of participation”. Moreover, in the initial stage of digital village development, any form of participation may represent a meaningful behavioral change regardless of the specific dimension it involved and may affect subjective wellbeing through social connections.

#### 3.2.3 Control variables

To mitigate potential confounding effects from other factors influencing rural residents’ happiness, this study incorporated a set of control variables based on prior research (Xu et al., 2026), covering three dimensions: characteristics of the household head, household characteristics, and village characteristics. Specifically, personal characteristics included the household head’s age, gender, marital status, and education. Household characteristics included household size and income. Village characteristics included terrain conditions and the distance from the village to the township government. Detailed definitions of all variables and their descriptive statistics are presented in Table 1.

**Table 1:** Variable definitions and descriptive statistics result.

Variables	Definitions and values	Mean	Std. dev.
Happiness	Are you happy with your current life? 1 = very unhappy; 2 = Not very happy; 3 = Average; 4 = Fairly happy; 5 = very happy	4.1525	0.8139
Digital village participation	Is there any digitization behavior in at least one of the following domains: digital infrastructure, digital economy, digital governance, or digital life? Yes = 1, No = 0	0.9366	0.2436
Digital infrastructure	How would you assess your household’s internet conditions? Good = 1, Poor = 0	0.8797	0.3253
Digital economy	Does your household engage in online transactions of products? Yes = 1, No = 0	0.0716	0.2580
Digital governance	Have you communicated with other villagers on important public affairs through WeChat groups? Yes = 1, No = 0	0.4968	0.5001
Digital life	Do you use mobile phones or the internet to meet your daily needs? Yes = 1, No = 0	0.5266	0.4994
Age	Age of household’s head (years)	54.4312	10.6396
Gender	1 = male 0 = female	0.9331	0.2498
Marriage	1 = married 0 = unmarried	0.9303	0.2547
Education	Household head’s level of education 1 = No formal education 2 = Primary school 3 = Junior high school 4 = High school 5 = Secondary vocational school 6 = Vocational high school 7 = Associate degree 8 = Bachelor’s degree 9 = Master’s/Doctoral degree	2.8493	1.0980
Family size	Total number of household members	4.1471	1.5177
Household income	Actual household income per year (in 10,000 CNY)	9.4837	28.0113
Terrain	1 = Plain 2 = Hills 3 = Mountainous 4 = Semi-mountainous	1.9277	0.9117
Distance	Distance from the village to the township government (Kilometer)	5.5167	5.4353

### 3.3 Research model

Based on the theoretical analysis presented above, this study establishes the following baseline regression model to examine the relationship between digital village participation and rural residents’ happiness:


$${\text{Happiness}}_{i}=\alpha_{0}+\alpha_{1}\text{Digital}\_{\text{village}}_{i}+\mu X_{i}+\varepsilon_{i}$$

Where the subscript i denotes the sample household; ${\text{Happiness}}_{i}$ represents the happiness level of the i -th rural resident; $\text{Digital}\_{\text{village}}_{i}$ represents the rural resident’s digital village participation level; $X_{i}$ denotes a set of control variables, including the household head’s personal characteristics, household characteristics, and village characteristics; $\varepsilon_{i}$ represents the random error term.

## 4 Results

### 4.1 Baseline regression results

A large number of existing studies have shown that estimation results obtained using ordered probit model, ordered logit model or OLS models generally do not differ significantly in terms of statistical significance or coefficient signs (Ferrer-i-Carbonell and Frijters, 2004; Zhong and Chen, 2022). Given that the dependent variable in this study is ordinal, an ordered probit or ordered logit model would be appropriate. The difference between the two models is that the error term of the former is assumed to follow a normal distribution, whereas that in the latter is assumed to follow a logistic distribution. To facilitate comparison with similar studies, this study reports the analysis results of the ordered probit model and conducts robustness tests using OLS and the ordered logit model.

Table 2 reports the estimation results for the association between digital village participation and rural residents’ happiness. Specifically, Column (1) presents the estimation results for overall digital village participation. The regression coefficient of digital village participation is 0.220 and is statistically significant at the 1% level, indicating a positive association between digital village participation and rural residents’ happiness. Columns (2)–Column (5) of Table 2 further report the effects of different dimensions of digital village participation on rural residents’ happiness. The results show that digital infrastructure, digital governance, and digital life all have significant positive effects on rural residents’ happiness, whereas the effect of the digital economy dimension is statistically insignificant.

**Table 2:** Baseline regression results for the effect of digital village participation on rural residents’ happiness.

Variables	(1)	(2)	(3)	(4)	(5)
	Happiness	Happiness	Happiness	Happiness	Happiness
Digital village participation	0.220^***^				
	(0.0811)				
Digital infrastructure		0.201^***^			
		(0.0606)			
Digital economy			−0.0600		
			(0.0777)		
Digital governance				0.246^***^	
				(0.0413)	
Digital life					0.338^***^
					(0.0422)
Age	0.0089^***^	0.0087^***^	0.0083^***^	0.0103^***^	0.0120^***^
	(0.0019)	(0.0020)	(0.0019)	(0.002)	(0.002)
Gender	−0.0138	−0.00839	−0.0133	−0.0223	−0.0329
	(0.0840)	(0.0841)	(0.0840)	(0.0841)	(0.0843)
Marriage	−0.0961^***^	−0.0946^***^	−0.0956^***^	−0.0936^***^	−0.109^***^
	(0.0324)	(0.0324)	(0.0324)	(0.0325)	(0.0325)
Education	0.0992^***^	0.0989^***^	0.103^***^	0.0866^***^	0.0822^***^
	(0.0194)	(0.0194)	(0.0194)	(0.0196)	(0.0196)
Family size	−0.0329^**^	−0.0312^**^	−0.0298^**^	−0.0320^**^	−0.0369^***^
	(0.0134)	(0.0134)	(0.0134)	(0.0134)	(0.0135)
Household income	0.0037^***^	0.0036^***^	0.0038^***^	0.0033^***^	0.0032^***^
	(0.001)	(0.001)	(0.001)	(0.001)	(0.001)
Terrain	−0.0526^**^	−0.0510^**^	−0.0583^***^	−0.0532^**^	−0.0392^*^
	(0.0222)	(0.0222)	(0.0221)	(0.0221)	(0.0222)
Distance	0.0043	0.0045	0.0042	0.0034	0.0057
	(0.0036)	(0.0037)	(0.0036)	(0.0036)	(0.0037)
Observations	3,126	3,126	3,126	3,126	3,126
Pseudo R2	0.0126	0.0131	0.0116	0.0165	0.0205

^*^p < 0.1, ^**^p < 0.05, ^***^p < 0.01.

### 4.2 Endogeneity test

Although this study controls for a series of demographic and socioeconomic variables, endogeneity may still exist in the relationship between digital village participation and rural residents’ happiness. This endogeneity may arise from two factors. First, omitted variable bias may arise because unobservable factors, such as individual psychological traits and social capital, may simultaneously influence digital participation and happiness. Second, reverse causality may occur if individuals with higher levels of happiness are more likely to adopt and engage with digital technologies. To address these endogeneity concerns, this study employs the two-stage least squares (2SLS) method to obtain more reliable estimates.

Following common practice, this part constructs two instrumental variables for the digital village participation index. First, based on the existing literature (Lei and Lin, 2009; Xie et al., 2023), we selected the average level of digital village participation among all farmers in the county, excluding those in the respondent’s own village, as the first instrumental variable. The average digital village participation level of other administrative villages within the county is strongly correlated with the digital village participation level of the respondent’s village, satisfying the relevance condition. After controlling for a series of individual-, household-, and village-level characteristics, the participation behavior of other villages is unlikely to directly affect the happiness of residents in the respondent’s village through other channels, thereby satisfying the exogeneity condition. Second, following Yu et al. (2020), we selected the internet penetration rate, defined as the average level of internet usage among farmers in other administrative villages within the county, as the second instrumental variable for digital village participation. This is because farmers’ participation in digital village practices is largely influenced by the level of local internet infrastructure. Higher internet penetration in other villages can significantly lower the digital participation threshold of residents in the respondent’s village through the demonstration effect and technological spillovers, thereby increasing their participation and satisfying the relevance condition. Moreover, internet usage among farmers in other villages is theoretically not directly related to the personal happiness of the surveyed farmers, thereby satisfying the exogeneity condition.

Table 3 reports the instrumental-variables estimation results. The first-stage regression results show that the F-statistic for the two instrumental variables are 50.3509 and 18.9936, respectively, indicating that the selected instruments do not suffer from weak instrument problems. Furthermore, in the first-stage estimation results, show that the instrumental variables are significantly associated with digital village participation at the 1% level, indicating strong instrumental relevance. The second-stage estimation show that the coefficient of digital village participation is positive and statistically significant at the 1% level. This indicates that, after accounting for endogeneity, digital village participation remains positively associated with farmers’ wellbeing. Meanwhile, the DWH test rejects the null hypothesis of exogeneity at the 1% level, suggesting that digital village participation is endogenous. Additionally, the *p*-value of the overidentification test is 0.8289, so the null hypothesis of instrument exogeneity cannot be rejected, indicating that the two instrumental variables satisfy the exogeneity condition.

**Table 3:** Endogeneity test results.

Variables	Instrumental variable 1		Instrumental variable 2	
	First stage	Second stage	First stage	Second stage
Digital village participation		1.6008^***^ (0.5177)		2.419^***^ (0.9293)
Instrumental variable	0.5672^***^ (0.0799)		0.2593^***^ (0.0594)	
Control variable	Yes	Yes		
Observations	3,126	3,126		
First stage F-statistic	50.3509^***^ (*p* = 0.0000)			18.9936^***^ (*p* = 0.0000)
Durbin Wu-Hausman test	9.3856^***^ (*p* = 0.0022)			8.679^***^ (*p* = 0.0032)
Overidentification test	*p* = 0.8289			

^***^p < 0.01.

### 4.3 Robustness test

#### 4.3.1 Replacement of the dependent variable

Life satisfaction refers to individuals’ subjective evaluations of their quality of life and is an important component of happiness. To test the robustness of the baseline findings, we replaced the dependent variable with life satisfaction. Column (1) of Table 4 reports the corresponding estimation results. The results show that digital village participation remains positively associated with rural residents’ happiness. Therefore, the findings confirm the robustness of the main conclusions.

**Table 4:** Robustness test.

Variables	(1)	(2)	(3)	(4)	(5)
	Replace the dependent variable	Replace the estimation model		Change the sample size	Replace the explanatory variable
		Ols	logit		
Digital village participation	0.217^***^	0.156^***^	0.397^***^	0.222^**^	0.178^***^
	(0.0809)	(0.0601)	(0.137)	(0.0872)	(0.021)
Age	0.0098^***^	0.0065^***^	0.0149^***^	0.009^***^	0.012^***^
	(0.0019)	(0.0014)	(0.0033)	(0.0023)	(0.002)
Gender	0.0633	−0.00853	−0.0482	−0.00960	−0.026
	(0.0829)	(0.0611)	(0.144)	(0.0881)	(0.084)
Marriage	−0.0523	−0.0750^***^	−0.159^***^	−0.116^***^	−0.100^***^
	(0.0324)	(0.0239)	(0.0566)	(0.0358)	(0.032)
Education	0.0675^***^	0.0743^***^	0.148^***^	0.113^***^	0.074^***^
	(0.0191)	(0.0138)	(0.0324)	(0.0201)	(0.019)
Family size	−0.0453^***^	−0.0233^**^	−0.0536^**^	−0.0287^**^	−0.037^***^
	(0.0133)	(0.00973)	(0.0229)	(0.0143)	(0.013)
Household income	0.0021^**^	0.0018^***^	0.0072^***^	0.0035^***^	0.003^***^
	(0.0009)	(0.0005)	(0.0024)	(0.001)	(0.001)
Terrain	−0.0146	−0.0439^***^	−0.0702^*^	−0.0520^**^	−0.038^*^
	(0.0220)	(0.0161)	(0.0377)	(0.0230)	(0.022)
Distance	−0.0017	0.0032	0.0072	0.0043	0.005
	(0.0036)	(0.0027)	(0.0062)	(0.0038)	(0.004)
Observations	3,126	3,126	3,126	2,923	3,126

^*^p < 0.1, ^**^p < 0.05, ^***^p < 0.01.

#### 4.3.2 Replacement of the estimation model

To further test the robustness of the results, we replaced the baseline probit model with OLS and ordered logit models. For ordered or continuous variables, the estimation results obtained from OLS and ordered logit models generally do not differ substantially from those of the probit model in terms of coefficient signs and statistical significance (Frijters et al., 2004). As shown in Columns (2) and (3) of Table 4, the estimated coefficient for digital village participation remains positive and statistically significant. These results further demonstrate the robustness of the findings.

#### 4.3.3 Restriction of the sample

To eliminate the potential influence of extreme values on the regression results and test the robustness of the estimates, we excluded observations for respondents aged over 70 years. As shown in Column (4) of Table 4, the sign and statistical significance of the estimated coefficients for digital village participation remain consistent with the baseline regression results, further supporting the robustness of the findings.

#### 4.3.4 Replace explanatory variable

To address the concern that binary variables might overlook differences in rural households’ participation levels of digital village participation, we re-estimated the main model using an alternative measure. We calculated the digital village participation score by summing the respondents’ score across the four questions. Higher values indicate higher levels of households’ participation. The regression results in Column (5) of Table 4 show that the positive impact of digital village participation on rural residents’ happiness remains significant, further verifying the reliability of the research results.

### 4.4 Mechanism test

#### 4.4.1 Alleviating credit constraints

Digital village participation enhances credit accessibility through digital financial tools, thereby alleviating economic constraints for rural residents. This strengthens their resilience to risk and sense of material security, thus laying the foundation for their happiness. In terms of credit constraints, although directly asking farmers whether they have ever been denied loans by banks is the ideal measurement method, such data are often unavailable in regular micro-surveys and are prone to recall bias or subjective concealment by respondents. Therefore, this study uses whether a household currently holds formal bank loans as a reverse proxy for credit constraints. The reason is as follows. Given that the penetration rate of formal financial services in rural areas is still relatively low, a farmer’s successful acquisition of a bank loan indicates that the household has effectively overcome the core obstacles in traditional rural credit markets, such as information asymmetry and insufficient collateral (Khan et al., 2024). Therefore, holding a formal bank loan indicates that credit constraints have been alleviated.

Table 5 presents the regression results. Specifically, columns (1)–(5) report the regression results regarding the effects of overall digital village participation and its sub-dimensions on credit constraints. It can be observed that there is a significant negative correlation between digital village participation and farmers’ credit constraints. The results of the four sub-dimension estimates show that digital economy, governance, and life have significant mitigating effects on residents’ credit constraints, whereas the coefficient of digital infrastructure is negative but statistically insignificant. Column (6) presents the baseline regression results, including only digital village participation and the control variables, whereas column (7) presents the results obtained by adding the mechanism variables to the model in column (6). As the coefficients in the table indicate, credit constraints have a significant negative effect on rural residents’ happiness. After including mechanism variables, the coefficient of the effect of digital village participation decreased. Furthermore, the 95% confidence interval from the bootstrap test (with 1,000 samples) did not include 0, indicating that the mediating effect was significant. Therefore, it can be concluded that digital village participation enhances residents’ happiness by alleviating credit constraints.

**Table 5:** Results of the mechanism analysis for credit constraint alleviation.

Variables	(1)	(2)	(3)	(4)	(5)	(6)	(7)
	Credit constraints					Happiness	Happiness
Digital village participation	−0.213^**^					0.220^***^	0.214^***^
	(0.101)					(0.0811)	(0.0812)
Digital infrastructure		−0.115					
		(0.0729)					
Digital economy			−0.415^***^				
			(0.0909)				
Digital governance				−0.158^***^			
				(0.0480)			
Digital life					−0.180^***^		
					(0.0491)		
Credit constraints							−0.0833^**^
							(0.0415)
Confidence interval of the Bootstrap test							[0.041,0.260]
Control variable	Yes	Yes	Yes	Yes	Yes	Yes	Yes
Observations	3,126	3,126	3,126	3,126	3,126	3,126	3,126

^**^p < 0.05, ^***^p < 0.01.

#### 4.4.2 Increase social interaction

By leveraging social platforms and online communities, digital village participation reconstructs rural social networks, strengthens emotional bonds, and facilitates information sharing, thereby mitigating social isolation and fostering a sense of belonging. This promotes psychological wellbeing by expanding social capital and providing emotional support. Regarding the proxy variables for social interaction, this study adopted the daily chatting and social interaction behaviors of rural residents as the core measure. This variable is measured by asking respondents whether they often engage in daily chatting or interaction with others through social platforms such as WeChat, Weibo, QQ, Zhihu, and Douban. The reasons for choosing this indicator are as follows. First, this variable directly measures social behaviors at the individual level and can accurately reflect the level of residents’ social interactions. Second, this variable focuses on the specific behavioral dimension of daily chatting and socializing and more precisely captures the core aspects of social interaction, namely emotional communication, information sharing, and relationship maintenance among individuals.

Table 6 presents the results. Specifically, columns (1)–(5) present the regression results regarding the effects of digital village participation and its various sub-dimensions on social interaction. It can be observed that there is a significant positive correlation between digital village participation and social interaction. The estimates across all four dimensions show that digital infrastructure, digital economy, digital governance, and digital life have a significant positive effects on rural residents’ social interactions. Column (6) presents the baseline regression results, including only digital village participation and the control variables, whereas column (7) presents the results obtained by adding the mechanism variables to the model in column (6). As the coefficients in the table indicate, social interaction has a significant positive effect on rural residents’ happiness. After the mechanism variables were included, the coefficient of digital village participation decreased. Furthermore, the 95% confidence interval obtained from the bootstrap test (with 1,000 samples) did not include 0, indicating that the mediating effect was significant. Therefore, this study argues that increased social interaction is one of the mechanisms through which digital village participation enhances rural residents’ happiness.

**Table 6:** Results of the mechanism analysis for social interaction.

Variables	(1)	(2)	(3)	(4)	(5)	(6)	(7)
	Social interaction					Happiness	Happiness
Digital village participation	0.500^***^					0.220^***^	0.193^**^
	(0.104)					(0.0811)	(0.0815)
Digital infrastructure		0.335^***^					
		(0.0830)					
Digital economy			0.633^***^				
			(0.181)				
Digital governance				1.099^***^			
				(0.0811)			
Digital life					0.365^***^		
					(0.0652)		
Social interaction							0.197^***^
							(0.0624)
Confidence interval of the Bootstrap test							[0.021,0.248]
Control variable	Yes	Yes	Yes	Yes	Yes	Yes	Yes
Observations	3,126	3,126	3,126	3,126	3,126	3,126	3,126

^*^p < 0.1, ^**^p < 0.05, ^***^p < 0.01.

### 4.5 The heterogeneous effects of the digital divide

#### 4.5.1 Effects of first-level digital divide

The first-level digital divide, or the “access divide”, refers to disparities in access to digital infrastructure and basic hardware among different groups. This divide centers on the distinction between “having” and “not having”, which directly determines whether an individual meets the basic threshold for digital village participation. This lack of physical access restricts the starting point for direct engagement, excludes some residents from the distribution of digital dividends, and potentially exacerbates inequality in happiness. In this study, the first-level divide is measured by whether rural residents own internet-enabled devices, such as mobile phones, tablets, and computers. The results in Columns (1) and (2) of Table 7 show that digital village participation significantly enhances residents’ happiness with internet-enabled devices, but has no significant effect on those without it. Although the coefficient difference between the two groups fails to reach statistical significance (*p* = 0.311), the positive effect observed in the access group remains pronounced, indicating that physical access is a prerequisite for digital participation to generate happiness gains.

**Table 7:** Results of the heterogeneous effects of the digital divide.

Variables	(1)	(2)	(3)	(4)	(5)	(6)
	Access divide		Skills divide		Outcomes divide	
	With access	Without access	High digital literacy	Low digital literacy	High satisfaction	Low satisfaction
Digital village participation	0.247^***^	−0.0102	0.481^**^	0.123	0.329^**^	0.136
	(0.0894)	(0.246)	(0.213)	(0.0893)	(0.141)	(0.0999)
Control variable	Yes	Yes	Yes	Yes	Yes	Yes
Observations	3,016	110	1,576	1,550	1,473	1,653
Inter-group coefficient difference	*p* = 0.311		*p* = 0.096		*p* = 0.212	

^*^p < 0.1, ^**^p < 0.05, ^***^p < 0.01. The inter-group difference test was conducted using the Seemingly Unrelated Regressions (SUR) method.

#### 4.5.2 Effects of second-level digital divide

The second-level digital divide goes beyond the physical access level and focuses on the “skills divide”, that is, differences in the knowledge and abilities individuals need to use digital technologies effectively. In the implementation of digital villages even when residents have access to internet connectivity and devices, they may still be unable to fully participate in online education, digital government services, or rural e-commerce due to a lack of digital literacy. In this study, the second-level digital divide was measured by the degree of difficulty respondents encountered when using internet-enabled devices. As shown in Columns (3) and (4) of Table 7, digital village participation has a significantly positive effect on happiness among respondents with a certain level of digital literacy. However, among the sample with lower digital literacy, the effect of such participation on happiness was positive but not significant. Furthermore, the *p* value of the inter-group coefficient difference test is 0.096, which is significant at the 10% statistical level. This indicates that, following access, the second-level digital divide further conditions the benefits of digital participation.

#### 4.5.3 Effects of the third-level digital divide

As a deeper dimension of the digital divide, the third-level digital divide emphasizes the “outcome divide”, which refers to the disparities in the actual benefits that different groups derive from using digital resources. Regarding digital village participation, even if residents have the necessary access and technical skills, the extent to which their happiness improves may vary depending on factors such as individual resource endowments, rural digital ecosystem support systems, and social networks. This study measured the third-level divide by the degree to which daily information needs are satisfied via the Internet. The results in columns (5) and (6) of Table 7 show that digital village participation has a significant positive effect on residents’ happiness at high satisfaction levels, whereas its effect at low satisfaction levels is not significant. Furthermore, the inter-group coefficient difference test showed that *p* = 0.212, which did not pass the significance level test. This indicates that although the influence directions of the two groups are different, the inter-group difference is not statistically significant. Nonetheless, the overall trend indicates that perceived effectiveness ultimately determines the extent to which happiness is achieved.

## 5 Discussion

This study found that digital village participation was generally positively associated with rural residents’ wellbeing, a result consistent with growing international evidence. This evidence, drawn from diverse national contexts, consistently reveals a positive relationship between digital technology adoption and subjective wellbeing (Fahmi and Sari, 2020; Ionescu-Feleagă et al., 2022). However, the impact of digital village development varies across different dimensions, particularly because the digitalization of the rural economy has no significant effect on happiness. Although numerous studies have shown that participation in the digital economy can significantly increase farmers’ income, promote entrepreneurship, and thereby enhance their subjective wellbeing (Zhao et al., 2024; Zheng and Ma, 2021), this study reached a different conclusion. In China, the levels of development of logistics networks, financial systems, and market integration, vary greatly across rural areas. Without the support of such conditions, the direct revenue-generating potential of digital economy participation may not be realized. Moreover, when economic gains reach a certain level, they may exacerbate the income gap within communities, thereby activating social comparison and weakening subjective wellbeing (Wu et al., 2024). When some families achieve significant economic success through digital channels while their neighbors fail to benefit in the same way, the resulting widening of income inequality may exacerbate the relative deprivation felt by those who lag behind. This has also been demonstrated in studies of happiness (Zhou et al., 2025). This finding suggests that policymakers should focus on the differentiated effects of different dimensions of digital participation rather than simply viewing digital participation as a homogeneous tool for enhancing happiness.

Through a progressive analysis of the three levels of the digital divide, this study further reveals that the positive effect of digital village participation on happiness is not uniformly distributed; instead, it is successively constrained by the three levels of the divide. Although the inter-group difference test did not reach the conventional significance level in some dimensions, the direction and trend of its impact still align with the theoretical expectations. This finding also has significant theoretical importance beyond the Chinese policy context. Specifically, the first layer, “access gap”, serves as a prerequisite: rural residents who have access to internet devices have a positive impact on their happiness through participation in digital village. The difference in the inter-group coefficients was not significant, possibly due to the limitations of the sample distribution (for example, the without access group had only 110 samples). However, this result also to some extent reflects the consensus on the fundamental role of digital access in studies of the digital divide (Wei et al., 2024). The conclusion of this study is in line with the assessment of the second-level digital divide in the existing literature. That is, the group with higher digital skills can obtain significant benefits from digitalization, while the group with relatively insufficient skills benefits less or not at all. This again confirms that digital skills are the key factor determining the distribution of digitalization benefits (Li et al., 2026). What is most notable is that our identification of the third layer, “the outcomes gap”, offers a unique perspective. The key to enhancing happiness lies in whether individuals can translate their digital participation to actual improvements in their living conditions. When individuals utilize digital technologies but fail to obtain meaningful benefits, they may become aware of their disadvantaged situation through upward social comparisons, thereby intensifying their subjective sense of relative deprivation and counteracting potential gains in happiness (Li et al., 2025). The inter-group coefficient difference test for the “outcomes gap” did not reach a significant level, but this “trend difference” still reveals the significant role of perceived effect at the psychological level. Although this finding was verified in rural areas of China, it also has implications for other developing countries undergoing digital transformation.

Despite its contributions, this study has several limitations. First, the effect of digital village participation on happiness may involve long-term dynamic effects that could not be captured in the temporal dimension of the current analysis. Future research could utilize multi-period longitudinal data to examine the effects of digital village participation more rigorously. Second, the measurement of digital village participation in this study reflected the average effect rather than the marginal effect of deepening digital participation. Future research using more detailed data (covering usage frequency, duration, digital skill levels, etc.) will help to reveal the differentiated impacts of participation intensity. Third, although the empirical analysis in this study has verified the two potential channels of alleviated credit constraints and enhanced social interaction, we also recognize that the underlying mechanism linking these two factors may be multidimensional and not limited to the analytical framework established in this study. Therefore, this study proposes two alternative paths worthy of further exploration in future research: the improving information acquisition capabilities and enhancing access to public services.

## 6 Conclusion and policy implications

Since the proposal of the “Implement the Digital Village Strategy”, rural China has been undergoing a profound digital transformation. Within this process, whether digital technology serves as a “bridge” leading rural residents toward a better life or creates a new “divide” due to disparities in access, skills, and the sense of gain remains an urgent empirical question. Using data from the 2020 China Rural Revitalization Survey (CRRS), this study systematically examined the impact of digital village participation on rural residents’ happiness, the underlying mechanisms, and the heterogeneous effects of the digital divide. The primary conclusions are as follows:

(1) Digital village participation is positively associated with rural residents’ happiness, although the effect varies across dimensions. The baseline regression results show that overall digital village participation has a positive effect on happiness, which is statistically significant at the 1% level. Digital village infrastructure, governance, and life all exert significant positive effects, whereas the digital village economy dimension failed to reach statistical significance. (2) Alleviating credit constraints and increasing social interaction are two critical transmission mechanisms through which digital participation bolsters happiness. (3) The heterogeneity test reveals that in the second-level (skills) divide, digital village participation has a more significant positive impact on the happiness of residents with higher digital literacy. Based on these findings, this study proposes the following policy recommendations.

First, it deepens the multidimensional construction of digital villages and enhances the precision of livelihood improvement. The impact of digital village construction on people’s happiness varies across dimensions. Policies should shift from all-round promotions to “all-round promotion” to “targeted measures”. On the one hand, efforts are being made to continuously consolidate the areas that have shown positive results, such as infrastructure, governance, and daily life, and to extend online government services and remote medical care to the village level. On the other hand, regarding the insignificant impact of the digital economy dimension, based on the empirical results of this study, we cannot assert that measures such as e-commerce training and the construction of logistics distribution systems will definitely be effective; however, it can reasonably be speculated that the insignificant effect in this dimension may be related to the low participation rate of e-commerce and the limited smoothness of the upward channel for agricultural products. Therefore, future policies implementing these measures should be accompanied by monitoring and evaluation to test their effectiveness.

Second, it establishes smooth mechanisms and enhances digital technology’s bridging role. Reducing credit constraints and promoting social interaction are two core transmission mechanisms. Policies should focus on maximizing the effectiveness of these two pathways. Efforts are being made in the financial sector to encourage agriculture-related financial institutions to utilize big data and blockchain technology to reduce information asymmetry, expand the coverage of credit loans, simplify the online loan process, and provide digital financial training for low-income farmers to ensure that they can access digital financial services. In terms of social interaction, village-level organizations are encouraged to utilize digital platforms to conduct online discussions and mutual assistance activities, thereby enhancing residents’ sense of community belonging and supporting the development of social applications suitable for the elderly to bridge the generation gap.

Third, we address the digital divide through stratification and build an inclusive digital village ecosystem. The three-level effect of the digital divide requires the implementation of stratified intervention strategies. In response to the “access divide”, efforts will be made to expand network coverage in remote mountainous areas, improved network speed, reduce fees, provide subsidies for terminal equipment, and ensure smooth connectivity at the “last mile”. To address the “skills divide,” a tiered training system has been established: the elderly focus on basic operations, while young adults receive advanced courses such as e-commerce operation, and college student village officials are organized to conduct scenario-based teaching in villages. Regarding the “outcome divide”, it is suggested that greater importance be attached to the feedback mechanism for the effectiveness of digital village participation. Through the collaborative governance of the three levels of the digital divide, digital technologies can truly become beneficial tools for all rural residents.

## Data Availability

Publicly available datasets were analyzed in this study. This data can be found at: http://rdi.cssn.cn/dcsj/.
